# Identification of poly(ADP-ribose)polymerase-1 and Ku70/Ku80 as transcriptional regulators of S100A9 gene expression

**DOI:** 10.1186/1471-2199-7-48

**Published:** 2006-12-22

**Authors:** Jens Grote, Simone König, Doreen Ackermann, Claudia Sopalla, Malgorzata Benedyk, Marek Los, Claus Kerkhoff

**Affiliations:** 1 Institute of Experimental Dermatology, University of Muenster, Germany; 2 Interdisciplinary Center for Clinical Research, Core Group Integrated Functional Genomics, Medical Faculty, University of Muenster, Muenster, Germany; 3 Manitoba Institute of Cell Biology, Winnipeg, Canada; 4 Interdisciplinary Center for Clinical Research (IZKF) Münster, Germany

## Abstract

**Background:**

S100 proteins, a multigenic family of non-ubiquitous cytoplasmic Ca^2+^-binding proteins, have been linked to human pathologies in recent years. Dysregulated expression of S100 proteins, including S100A9, has been reported in the epidermis as a response to stress and in association with neoplastic disorders. Recently, we characterized a regulatory element within the S100A9 promotor, referred to as MRE that drives the S100A9 gene expression in a cell type-specific, activation- and differentiation-dependent manner (Kerkhoff et al. (2002) J. Biol. Chem. 277, 41879–41887).

**Results:**

In the present study, we investigated transcription factors that bind to MRE. Using the MRE motif for a pull-down assay, poly(ADP-ribose)polymerase-1 (PARP-1) and the heterodimeric complex Ku70/Ku80 were identified by mass spectrometry and confirmed by chromatin immunoprecipitation. Furthermore, TPA-induced S100A9 gene expression in HaCaT keratinocytes was blocked after the pharmacologic inhibition of PARP-1 with 1,5-isoquinolinediol (DiQ).

**Conclusion:**

The candidates, poly(ADP-ribose)polymerase-1 (PARP-1) and the heterodimeric complex Ku70/Ku80, are known to participate in inflammatory disorders as well as tumorgenesis. The latter may indicate a possible link between S100 and inflammation-associated cancer.

## Background

Members of the S100 protein family comprise a multigenic group of non-ubiquitous cytoplasmic Ca^2+^-binding proteins of the EF-hand type, differentially expressed in a wide variety of cell types. In particular, S100A8 and S100A9 also known as calgranulins are abundant in myeloid cells. The expression of S100A8 and S100A9 is increased in various disorders, such as rheumatoid arthritis, inflammatory bowel disease and vasculitis [1]. The S100/calgranulins are associated with inflammatory disorders as they are secreted from phagocytes upon cellular activation [2,3], and track disease activity. In addition to their abundance in myeloid cells, S100A8 and S100A9 can also be found in the epidermis as a response to stress. For example, they are significantly up-regulated in differentiating suprabasal wound keratinocytes [4,5], in response to UVB irradiation [6], and in psoriasis keratinocytes [7], suggesting a role for these proteins in the pathogenesis of certain diseases. Based on these findings, the two S100 proteins have been referred to as stress-regulated proteins [8].

An additional important indication for their involvement in inflammatory and neoplastic disorders is that most S100 genes are found near a region on human chromosome 1q21 which is responsible for a number of chromosomal abnormalities [9,10]. This results in a dysregulated expression of S100A9 as well as other S100 genes associated with neoplasias [11]. Although the function of S100 proteins in cancer cells in most cases is still unknown, the specific expression patterns of these proteins is a valuable prognostic tool. In addition, a psoriasis susceptibility region, the PSORS4 locus, is mapped to chromosome 1q21 [12].

Despite a number of distinct regulatory regions located upstream of the transcription initiation site that are known to either activate or repress promoter activity of S100 genes in a differentiation and tissue/cell-specific manner, the corresponding nuclear factors as well as the underlying molecular mechanisms still remain unclear [13]. In addition to PU.1 [14], C/EBP-α and -β [15] have been shown to drive S100A9 gene expression in the myeloid lineage. Recently, cytokine oncostatin M (OM) has been demonstrated to strongly induce the S100A9 gene expression [16]. Promoter analysis provided evidence that S100A9 represents a novel OM-regulated gene acting through the STAT3-signaling cascade. This finding is in accordance with another study showing that IL-22 up-regulates the expression of S100A7, S100A8, and S100A9 in keratinocytes since IL-22 induces STAT3 activation in keratinocytes [17].

In a previous study, we identified a regulatory element within the S100A9 promoter referred to as MRP regulatory element (MRE) that drives the S100A9 gene expression in a cell-specific and differentiation-dependent manner [13]. This regulatory region is located at position -400 to -374 bp, and two distinct nuclear complexes were demonstrated to bind to this region. Interestingly, the formation of the nuclear protein complexes closely correlates with the myeloid-specific expression of the S100A9 gene and, were therefore referred to as MRE-binding complex A (MbcA) and MbcB, respectively. Analysis of one of the two nuclear complexes revealed a heterocomplex consisting of transcriptional intermediary factor 1β (TIF1β) and a yet unidentified protein with homology to KRAB domain-containing (Kruppel-related) zinc finger proteins (ZFP) [13].

In order to identify the other nuclear complex we performed DNA affinity chromatography studies employing MRE oligonucleotides as an affinity matrix. Further extensive investigations provide strong evidence that a complex of PARP-1, Ku70 and Ku80 was involved in the regulation of the S100A9 gene expression. This finding was confirmed by chromatin immunoprecipitation (ChIP) analysis.

## Results and discussion

### Identification of MRE-binding proteins

**Figure 1:**
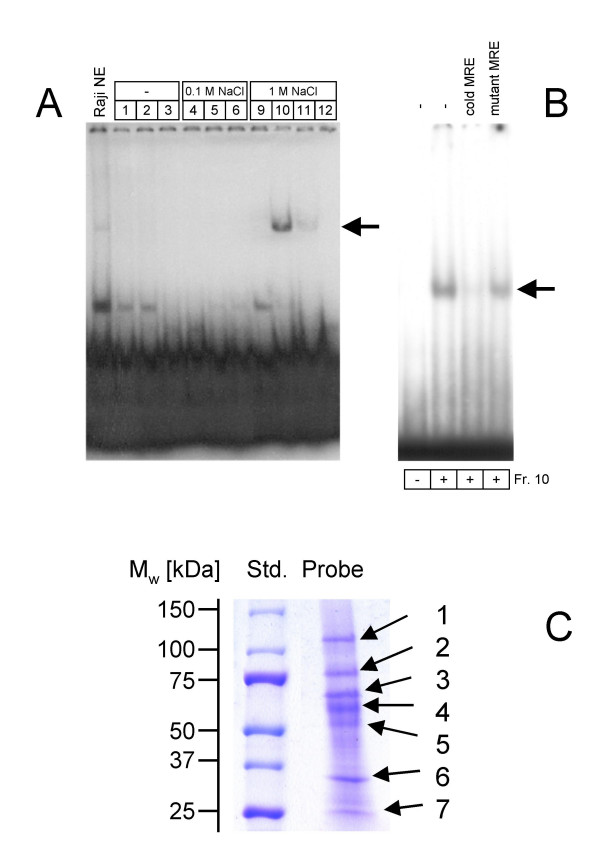
**(A) DNA affinity chromatography**. Nuclear proteins (20 mg total protein) were prepared from Raji cells, and subjected to DNA affinity chromatography with the biotinylated MRE oligonucleotide (position -400 to -357 bp). Proteins specifically bound to MRE were eluted with different NaCl concentrations, and 10 μl-aliquots (800 μl total) were analyzed by EMSA. **(B) Competition EMSA analysis**. For competition analysis of MRE-binding, either unlabelled MRE oligonucleotide or MRE mutant oligonucleotide was added at a 100-fold molar excess to the binding reactions. **(C) Separation of the proteins bound to MRE**. The 1M NaCl eluate of affinity chromatography was precipitated with UPPA-Protein Concentrate Kit according to the manufacturer's protocol. The precipitate was dissolved in SDS-PAGE sample buffer and subjected to SDS-PAGE (Ready-Gel gradient gel 4–15% (Biorad, Munich, Germany). The proteins were stained by Coomassie R250 The proteins are numbered according their molecular weights.

In order to identify the proteins participating in the formation of the complex we performed DNA affinity chromatography. Nuclear extracts of Raji cells were subjected to affinity purification employing MRE oligonucleotides as an affinity matrix. Nuclear proteins bound to MRE were eluted in a stepwise gradient with 0, 100, and 1,000 mM NaCl and then analyzed by EMSA. Only the proteins of the 1 M NaCl eluate displayed DNA-binding activity, indicating that they specifically interacted with the probe (Fig. 1A). The binding of proteins to MRE was specific, as an excess of non-labeled MRE oligonucleotide efficiently competed with the labeled probe in complex formation, whereas the mutant oligonucleotide MRE (T386C,G385T,A380C,A379C) did not compete (Fig. 1B). Our previous study showed that this MRE mutant oligonucleotide was unable to compete for binding to MRE oligonucleotide [13].

Then the proteins of the 1 M NaCl eluate were precipitated using the UPPA Concentrate Kit, then subjected to SDS-PAGE, and finally visualized by Coomassie staining (Fig. 1C).

Seven protein bands with apparent molecular weights of 110, 75, 65, 60, 35, and 25-kDa were detected. The protein bands were excised from the gel, digested with trypsin, and subjected to MALDI-MS for peptide mass fingerprint and to ESI-MS/MS for peptide sequencing. The data obtained were analyzed using Swissprot and NCBI databases. The identified proteins are summarized in Table 1.

**Figure 2:**
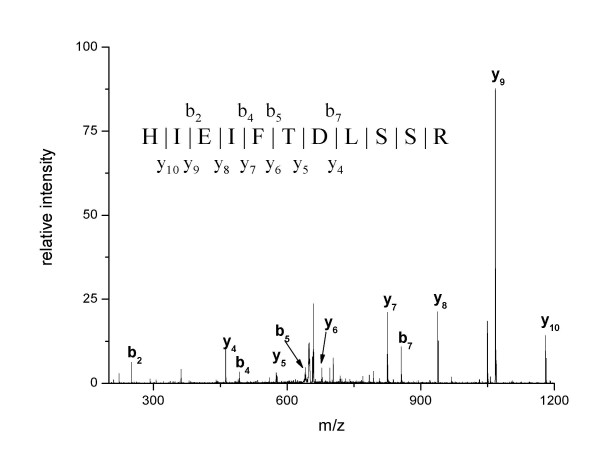
**MS/MS spectrum of [M+2H]^2+ ^at m/z = 750.4 assigned to peptide AA 66–78 of PARP-1 measured in the digest of band 7**. Ion series y and b were labeled according to [54].

**Figure 3:**
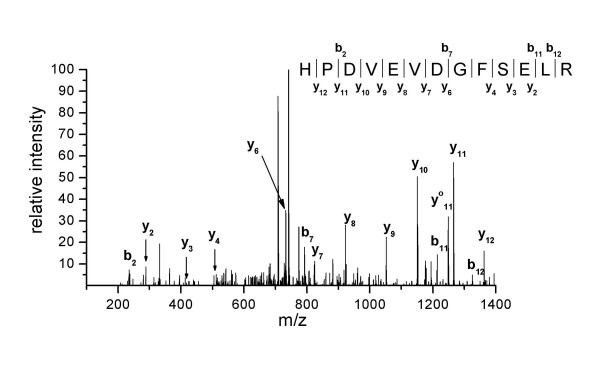
**MS/MS spectrum of [M+2H]^2+ ^at m/z = 659.34 assigned to peptide AA 131–141 of Ku80 measured in the digest of band 2**. For further details see legend to Figure 2.

Specifically, PARP-1 (113 kDa) was identified in band 1 by peptide mass fingerprint and peptide sequencing (Tab. 2). Fragments of PARP-1 were found in nearly all bands, especially in band 7 (Fig. 2). Ku80 (83 kDa) was identified in band 2 (Tab. 3** and **Fig. 3) and Ku70 (70 kDa) in band 3 by peptide mass fingerprint and peptide sequencing (Tab. 4). The identity of PARP-1 and Ku80 was also confirmed by Western Blotting (**see **Additional file 1).

PARP-1 represents a nuclear enzyme with a DNA nick sensor function [18]. Upon binding to broken DNA, PARP becomes activated and cleaves NAD^+ ^into nicotinamide and ADP-ribose. It then polymerizes ADP-ribose on nuclear acceptor proteins including histones, transcription factors, and PARP itself [18]. Poly ADP-ribosylation has been implicated in a number of cellular processes, such as DNA repair [19], transcriptional regulation [20-22], and cell cycle progression [22]. Excessive PARP activation induced by oxidative stress has been shown to decrease cellular NAD^+ ^and ATP pools, resulting in cell dysfunction or necrotic cell death [23-25]. The PARP activation pathway contributes to tissue injury in various forms of shock, inflammation, trauma, and reperfusion injury, as indicated by the protection provided by PARP inhibitors or the PARP^-/- ^phenotype in these disease models [25].

Both Ku80 and Ku70 form the heterodimeric DNA binding complex Ku that was originally identified two decades ago as a major target of autoantibodies in Japanese patients with the scleroderma-polymyositis overlap syndrome [26]. The autoantibodies against Ku have since been found in subsets of patients suffering from a number of different autoimmune diseases, including systemic lupus erythematosus and scleroderma.

Ku is best known for its crucial role in DNA repair [27]. Ku is involved in both major pathways of DNA double strand break repair: homologous recombination and non-homologous end joining. Besides its vital role in DNA repair, reports have implicated Ku in certain other cellular processes, including telomere maintenance, antigen receptor gene arrangements, cell cycle regulation, regulation of heat shock-induced responses, and specific gene transcription and apoptosis. For example, it has been shown that Ku is required for interleukin-13/-4-induction of 15-lipoxygenase-1 gene expression in human epithelial cells [28]. Here, Ku is induced in response to IL-13 and IL-4, and a 29 bp region within the -353 to -304 bp region of the 15-LO-1 promoter is required for its binding and subsequent induction of 15-LO-1 gene expression.

Studies with cells and/or animals deficient in Ku have shown that dysfunction and dysregulation of the above-mentioned processes lead to tumor development [27]. In addition, a rare microsatellite polymorphism in the Ku80 gene is associated with cancer in patients with varying radiosensitivity [29].

Since PARP-1 and Ku bind to DNA double strand breaks we performed DNA affinity chromatography employing the MRE mutant oligonucleotide MRE (T386C,G385T,A380C,A379C) as affinity matrix in order to verify whether this binding is specific to the MRE sequence. Our previous study showed that this MRE mutant oligonucleotide was unable to compete for binding to MRE oligonucleotide [13]. We failed to detect DNA-binding activity in the corresponding 1 M NaCl eluate, and after protein precipitation, no protein bands were visualized by Coomassie staining (**data not shown**), indicating that the high affinity of PARP-1 and KU to DNA double strand breaks was not responsible for their binding to the MRE oligonucleotide.

### ChIP experiment

**Figure 4:**
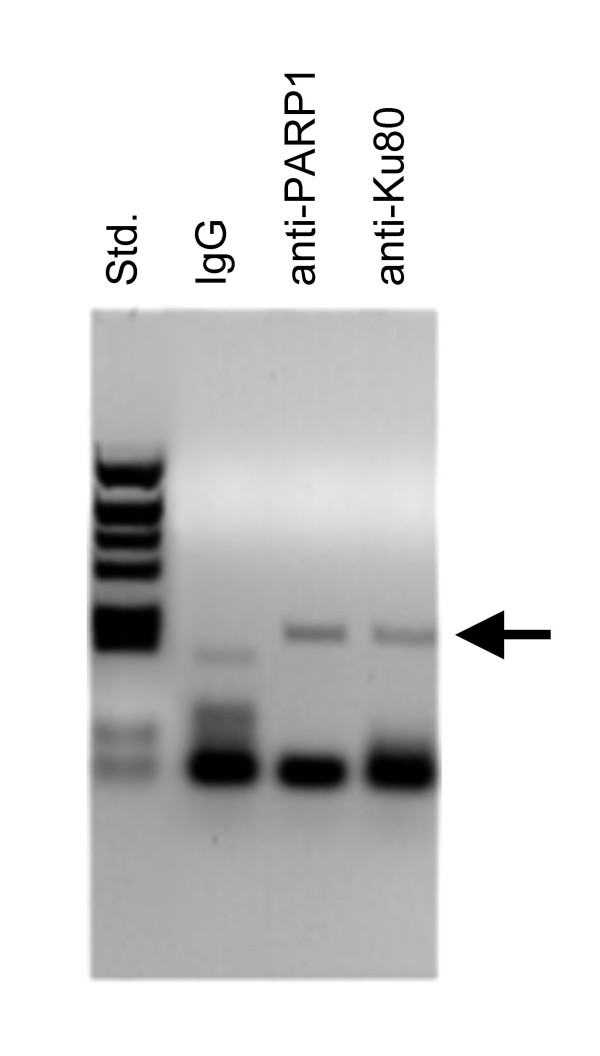
**ChIP with specific antibodies against PARP-1 and Ku80**. ChIP was performed on chromatin from Raji cells, using antibodies as indicated. PCR was performed using primers specific for the S100A9 promoter as described in the methods section.

To verify whether PARP-1 and Ku80 can directly bind to the S100A9 promoter in vivo, we performed chromatin immunoprecipitation. Cross-linked chromatin from Raji cells was immunoprecipitated with specific antibodies against PARP-1, Ku80, and the nonspecific antibodies IgG. After PCR analysis using S100A9 promoter-specific oligonucleotides, we found that PARP-1 and Ku80 specifically bind the regulatory region of S100A9 promoter. Chromatin immunoprecipitates with nonspecific IgG antibodies did not reveal any specific binding to the analyzed regulatory regions (Fig. 4).

Thus, the ChIP experiments clearly confirmed that both PARP-1 and Ku80 specifically bind to MRE. It is likely that PARP-1 and Ku80 (together with Ku70) are components of the nuclear complex that binds to MRE. This assumption is supported by several reports demonstrating the interaction of PARP-1 with Ku proteins. A complex formed by PARP-1, Ku proteins and C/EBPα has been shown to be involved in the increasing sensitivity of prostate cancer cells to DNA damage [30].

**Figure 5:**
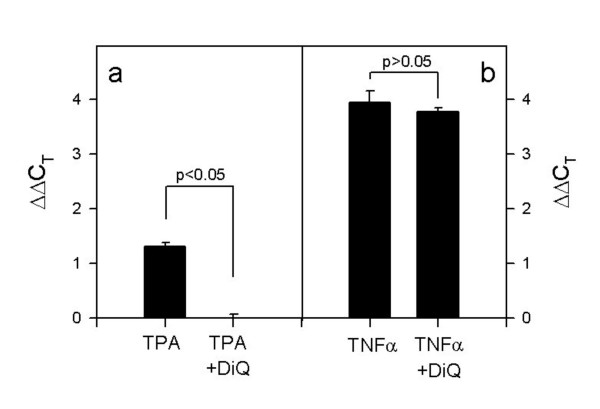
**S100A9 gene expression in HaCaT cells after TPA (A) and TNFα (B) stimulation**. HaCaT cells were stimulated with either 100nM TPA or 50 ng/ml TNFα in the presence and absence of DiQ (20 μM) for 18 h. Then total RNA was extracted from the cells, and S100 gene expression analysis was performed by quantitative PCR analysis. Relative S100 gene expression is expressed in relation to the corresponding S100 gene expression in non stimulated cells: ΔΔC_T _S100 (stimulated) = ΔC_T _S100 (stimulated) – ΔC_T _S100 (non-stimulated). The number of experiments was n = 3.

Strong evidence that PARP-1 is involved in S100A9 gene regulation has also recently reported by Martin-Oliva and co-workers [31]. They showed that TPA-induced S100A9 gene expression was blocked after the pharmacologic inhibition of PARP-1 with 3,4-dihydro-5- [4-(1-piperidinyl)butoxyl]-1(2H)-isoquinolinone. In accordance with this finding, they also did not observe TPA-induced S100A9 gene expression in *parp1 *knockout mice. Therefore, we investigated S100A9 gene expression in HaCaT keratinocytes in the absence and presence of the PARP-1 inhibitor 1,5-isoquinolinediol (DiQ). Both TPA- and TNFα- stimulation resulted in increased levels of S100A9 mRNA transcripts. S100A9 gene expression was n^1.45^= 2.7-fold increased after TPA treatment and n^3.94^= 15.3-fold increased after TNFα stimulation, respectively (Fig. 5). While the TPA-increased S100A9 gene expression was completely abrrogated by DiQ, TNFα-induced S100A9 gene expression was not affected by DiQ. Together with the report of Martin-Oliva and co-workers [31], these findings indicate the involvement of PARP-1 in S100A9 gene regulation.

Further correlations of PARP-1 and S100A9 gene expression are found in other reports: Dazard and co-workers [6] reported that in addition to other genes, S100A9 gene expression is up-regulated in normal human epidermal keratinocytes in response to ultraviolet B radiation (UVB). Induction of S100A9 gene expression is also found in epithelial cells in specific pathophysiological conditions, such as wound healing [4,5] and psoriasis [7]. Similarly, PARP-1 activity is increased after UV irradiation and in psoriasis [32]. Our finding that PARP-1 regulates the S100A9 gene expression represents the first link between this nuclear protein and the S100 protein family.

What is the molecular mechanism by which PARP-1 regulates S100A9 gene expression?

Roles for PARP-1 in the transcriptional regulation of specific genes have been demonstrated in several physiological contexts [33-35]. From these studies, at least two different mechanisms have been proposed for the regulation of transcription by PARP-1: (1) modulating chromatin structure, and (2) acting as part of gene-specific enhancer/promoter-binding complexes. As a component of enhancer/promoter-binding complexes, PARP-1 acts to stimulate transcription with some activators, while inhibiting transcription with others, depending on the cell type and promoter context. In some cases, PARP-1 enzymatic activity is not required for its transcriptional co-regulator function (e.g., with NF-κB). Recently, Ju and co-workers [36] have presented some mechanistic details of how PARP-1 acts as a modulator of the chromatin structure: In complex with DNA topoisomerase IIβ (TopoIIβ), PARP-1 is recruited to specific promoter regions. Then, transient dsDNA break formation mediated by TopoIIβ induces PARP-1 enzymatic activity, which is required for a nucleosome-specific histone H1-high-mobility group B exchange event and for local changes of chromatin architecture. The authors conclude that TopoIIβ/PARP-1 complex-mediated transient dsDNA breaks serve as a general mechanism for regulated initiation of gene transcription by nuclear receptors and other classes of DNA binding transcription factors, including AP-1.

AP-1 has been assumed to be involved in S100A9 gene expression [31,37,38], and poly(ADP ribosyl)ation of AP-1 by PARP-1 appears to be important for its binding activity as shown by Martin-Oliva and co-workers [31]. Pharmacological inhibition of PARP-1 results in significant reduction of the relative levels of AP-1-binding activity compared to mice treated with TPA [31]. In contrast, NF-κB activation was not reduced by PARP-1 inhibitors but reduced in parp-1^-/- ^cells [31,39,40]. It has been reported that in addition to NF-κB, AP-1 is also induced by TNFα receptor ligation [41]. In the present study, however, we demonstrated that TPA-increased S100A9 gene expression was blocked by the PARP-1 inhibitor, while TNFα-increased S100A9 mRNA level was not affected. These findings might indicate that both interaction of PARP-1 with transcription factors and PARP-1 mediated poly(ADP ribosyl)ation are important for S100A9 gene expression.

It is noteworthy to point to the association of S100 proteins with tumorgenesis. An increasing body of evidence provided by several reports shows that S100 gene are differentially regulated in various cancer cells [16,42-53]. PARP-1 and Ku have also been assigned a role in tumorgenesis. Therefore, this study provides first insights into the molecular mechanism of S100A9 gene regulation, and it opens an avenue for a better understanding as to why S100 gene expression is associated with tumor promotion. Nevertheless, we are aware that the exact elucidation of the DNA/protein complexes will require further intensive investigations.

## Conclusion

The observations reported here demonstrate the role of PARP-1 (and probably Ku70/80) in S100A9 gene regulation. The association of S100 proteins with inflammatory and neoplastic disorders together with the role of PARP-1 in stress response and tumorgenesis might indicate the link between S100 and inflammation-associated cancer.

## Methods

### Nuclear Extraction and Electrophoretic Mobility Shift Assays (EMSAs)

Nuclear extracts of Raji cells were essentially prepared as described earlier [13]. For the EMSA reaction, a double-stranded oligonucleotide coding for bp -400 to -357 of the S100A9 promoter was used. The sense oligonucleotide CAGACCATCCTTGTTGGACTAAAAGGAAGGGGCAGACTGCCATG and its antisense strand CATGGCAGTCTGCCCCTTCCTTTTAGTCCAACAAGGATGGTCTG were annealed and end-labeled by T_4 _polynucleotide kinase and [γ-^32^P] ATP (Hartmann Analytic, Braunschweig, Germany). EMSAs were performed with nuclear extracts as follows: nuclear protein (50 *μ*g) was mixed with 3 *μ*g of sheared genomic salmon sperm DNA and 100,000 cpm of the labeled probe (approximately 1 ng) in EMSA buffer [20 mmol/L Hepes (pH 7.5), 1 mmol/L MgCl_2_, 75 mmol/L KCl, 1 mmol/L DTT, 0.018% (v/v) Nonidet P-40] in a total volume of 36 *μ*l. This mixture was allowed to incubate for 60 min at 4°C. Samples were mixed with 12 *μ*l of sample buffer [50% (w/v) sucrose, 0.5 × TBE (where 1 × TBE buffer is 90 mmol/L Tris/borate, 2 mmol/L EDTA, pH 8.0)] and then run on a non-denaturing 5% polyacrylamide gel in 0.25 × TBE buffer. The gels were dried and exposed to Kodak XAR-5 X-ray film.

For competition analysis of MRE-binding, either unlabelled MRE oligonucleotide or a MRE mutant oligonucleotide was added at a 100-fold molar excess to the binding reactions.

### DNA affinity chromatography

The biotinylated MRE oligonucleotides were synthesized by Applied Biosystems Oligo Factory (Weiterstadt, Germany). For DNA affinity purification, 5–20 mg nuclear protein was incubated with 4 nmol double-stranded, biotinylated MRE oligonucleotide and 570 μg sheared genomic salmon sperm DNA in 6,000 μl EMSA-buffer. After rotating the samples for 1 hour at 4°C, 4 ml Immunopure Immobilizied Streptavidin (Pierce Biotechnology, Rockford, IL, USA) was added and further incubated for 1 h at 4°C. Then the sample was applied to an empty PD10 column and the proteins bound to MRE beads were eluted with EMSA-buffer supplemented with 0, 100, and 1,000 mM NaCl. Aliquots of the different fractions were analyzed by EMSA as well as subjected to SDS-PAGE using standard protocols.

### Protein concentration and SDS-PAGE

The affinity chromatography eluate (460 μl) was precipitated with UPPA-Protein Concentrate™ Kit (Geno Technology, Inc., St. Louis, MO, USA) according to the manufacturer's protocol. The precipitate was dissolved in SDS-PAGE sample buffer (0,125 M Tris at pH 6,8, 4% SDS, 10% (v/v) β-mercaptoethanol, 20% (v/v) glycerol, 0,02% (w/v) bromophenol blue). SDS-PAGE (Ready-Gel gradient gel 4–15% (BioRad, Munich, Germany) was performed with running buffer using standard protocols. The gel was stained with Coomassie R250 (4% (w/v) in 44% methanol, 9.2% acetic acid).

### Protein digestion and Mass Spectrometry (MS)

Stained protein bands were excised, destained in 25 mM ammonium bicarbonate containing 50% methanol, rinsed with acetic acid/methanol/water 10/45/45 (v/v/v) and then shrunk with acetonitrile and dried. Digestion solution (30 ng/μl trypsin sequencing grade (Roche, Penzberg, Germany) in 10 mM ammonium bicarbonate at pH 9) was applied to each gel piece until swelling was complete (i.e. 10–15 μl depending on gel size). Digestion buffer was added to cover the gel pieces. After overnight digestion at 37°C, peptides were extracted using increasing concentrations of acetonitrile containing 5% formic acid (50%, 80%, 100% subsequently) and desalted with μC18 ZipTips (Millipore, Bedford, MA, USA) according to the manufacturer's protocol.

For peptide mapping, a matrix assisted laser desorption/ionization time-of-flight (MALDI-TOF) instrument TofSpec 2E (Waters/Micromass, Manchester, UK) was used in positive ion reflectron mode. 0.5 μl of peptide mixture and 0.5 μl of 4-hydroxy-α-cyano cinnamic acid (10 mg/ml in 50% acetonitrile and 0,05% trifluoro acetic acid) were spotted and mixed on the MALDI target. For measurement, ion suppression was set to 500 and spectra were externally calibrated and internally corrected by trypsin autolysis peaks.

For peptide sequencing with collision induced dissociation an electrospray ionization (ESI) ion trap mass spectrometer Esquire3000 (Bruker Daltonics, Bremen, Germany) was used. For manual nanospray measurements a modified liquid-junction ion source and home-made omega-glass capillaries were employed. Digestion was measured in 50% methanol/5% acetic acid. For HPLC-MS/MS, peptides were separated on a 75 μm ID C18 column (PepMap, LC Packings, Amsterdam, The Netherlands) using a 30 min gradient from 5% to 100% solvent B (solvent A: 0.05% formic acid/acetonitrile (95/5, v/v), solvent B: 0.04% formic acid/acetonitrile (20/80, v/v)) at a flow rate of 250 nl/min.

Peptide mass fingerprints and sequence data were searched against the databases SwissProt (Swiss Institute of Bioinformatics) and NCBI (National Center for Biotechnology Information, USA) employing the Mascot search engine (Matrix Science LTD, London, UK).

### Formaldehyde cross-linking and chromatin immunoprecipitation

DNA and proteins were cross-linked by the addition of formaldehyde (1% final concentration) 10 min before harvesting, and cross-linking was stopped by the addition of glycine pH 2.5 (125 μM final concentration) for 5 min at room temperature. Cells were centrifuged, resuspended in hypotonic buffer, and passed through a 26-gauge needle. Nuclei were spun down, resuspended in 300 μl of SDS lysis buffer (1% SDS, 10 mM EDTA, 50 mM Tris-HCl, pH 8, and a protease inhibitor mixture), and sonicated to generate 500-2000 bp fragments. After centrifugation, the cleared supernatant was diluted 10-fold with immunoprecipitation buffer (50 mM Tris-HCl, pH 8, 150 mM NaCl, 5 mM EDTA, 0.5% Nonidet P-40). The cell lysate was precleared by incubation at 4°C with 15 μl of protein G beads preadsorbed with sonicated single-stranded DNA and bovine serum albumin. The cleared lysates were incubated overnight with polyclonal antibodies against PARP-1 and Ku80, or with nonspecific IgG antibody. Immune complexes were precipitated with 30 μl of protein G beads preadsorbed with sonicated single-stranded DNA and bovine serum albumin. After centrifugation the beads were washed, and the antigen was eluted with 1% SDS and 100 mM sodium carbonate. DNA-protein cross-links were reversed by heating at 65°C for 4–5 h, and DNA was phenol-extracted and ethanol-precipitated. Then PCR analysis was performed using S100A9 promoter-specific oligonucleotides. For PCR analysis, oligonucleotides encompassing bp -435 to -415 (forward primer: 5'-AGTATCACAGAGCCAGGCAAG-3') and bp -215 to -196 (reverse primer:5'-GTTTGCAGGAAGCTGGTTGT-3') of the S100A9 promoter was used.

### Quantitative PCR

HaCaT cells were stimulated as indicated. Then total RNA was extracted from cells using the RNA Isolation Kit (Qiagen, Germany) and first strand cDNA was synthesized according to common molecular biology techniques. The level of mRNA transcripts for S100A9 was estimated by real-time PCR using forward primer 5'- GGAATTCAAAGAGCTGGTGCG-3'and reverse primer 5'-GCATTTGTGTCCAGGTCCTCC-3'. C_T _values of target genes were normalized to GAPDH. Relative S100 gene expression is expressed in relation to the corresponding S100 gene expression in non stimulated cells: ΔC_T _S100 (stimulated) – ΔC_T _S100 (non-stimulated) = ΔΔC_T _S100 (stimulated).

## Abbreviations

DiQ, 1,5-isoquinolinediol; ESI, electrospray ionization; HPLC, high performance liquid chromatography; MALDI, matrix-assisted laser desorption/ionization; MRE, mrp14 promoter regulatory element; MS, mass spectrometry; TOF, time-of-flight; TopoIIβ, DNA topoisomerase IIβ

## Authors' contributions

JG carried out the identification of the MRE-binding proteins and helped to draft the manuscript. SK participated in the mass spectrometry analysis and helped to draft the manuscript. DA participated in the mass spectrometry analysis. CS and MB carried out the ChIP and quantitative PCR analysis. ML helped to draft the manuscript. CK participated in the identification of the MRE-binding proteins, conceived the study, and drafted the manuscript. All authors have read and approved the final manuscript.

## Supplementary Material

Additional File 1 **Western Blot analysis.** Aliquots of the Raji nuclear extract (NE) and the 1 M NaCl eluate of affinity chromatography (M) was subjected to SDS-PAGE. The Western-blot analysis was performed using PARP-1- and Ku80-specific antibodies. Click here for file

## Figures and Tables

**Table 1:** Summary of proteins identified by peptide mass fingerprint and peptide sequencing

**Band (molecular weight)**	**Proteins, abbreviation, and SwissProt accession number**	**Number of sequenced peptides**
1 (110 kDa)	- Poly (ADP-ribose) polymerase 1 (PARP-1; P09874)	3 (m)
2 (75 kDa)	- ATP-dependent helicase II (Ku80; P13010)	1 (m)
3 (65 kDa)	- ATP-dependent helicase II (Ku70; P12956)	2 (m)
4 (60 kDa)	- PARP-1 - Ku70 - Keratin	2 (LC) 2 (LC)
5 (50 kDa)	- PARP-1 - Keratin	6 (LC)
6 (35 kDa)	- DNA-(apurinic or apyrimidine site) lyase (APEX1; P27695) - Glyceraldehyde-3-phosphate hydrogenase (GAPDH; P04406)	9 (LC) 3 (LC)
7 (25 kDa)	- PARP-1	9 (LC)

Labels "m" refer to verification by manual ESI-MS/MS and "LC" to HPLC-MS/MS, respectively.

**Table 2:** MALDI-MS results of PARP-1

**PARP-1**		
Amino acid residues		[M+h]+
**1–15**	**MAESSDKLYRVEYAK**	**1700.85 (AcetN)**
66–78	HPDVEVDGFSELR	1499.71
109–119	TLGDFAAEYAK	1185.57
109–122	TLGDFAAEYAKSNR	1542.76
168–182	EEL GFRPEYSASQLK	1753.88
332–340	EWVTPKEFR	1191.62
487–496	AEPV EVVAPR	1066.58
637–654	KFYPLEIDYGQDEEAVKK	2172.09
638–654 or 637–653	FYPLEIDYGQDEEAVKK KFYPLEIDYGQDEEAVK	2043.99
685–700	AMVEYEIDLQKMPLGK	1896.95 (2 MetOx)
848–858	YKP FKQLHNRR	1484.72
858–865	RLL WHGSR	1024.58
**866–878**	**TTNFA GILSQGLR**	**1377.75**
**879–893**	**IA PPEAPVTGYM FGK**	**1593.80 (MetOx)**

Peptides in bold were sequenced with MS/MS on the doubly charged ion. MetOx refers to peptides with oxidized methionines.

**Table 3:** MALDI-MS results of Ku80

**Ku80**		
Amino acid residues		[M+h]+
36–44	KVITMFVQR	1137.65 (1MetOx)
37–44	VITMFVQR	1009.55 (1MetOx)
**131–141**	**HIEIFTDLSSR**	**1317.68**
354–363	RFFMGNQVLK	1255.66 (1MetOx)
355–363	FFMGNQVLK	1099.56 (1MetOx)
401–413	ANPQVGVAFPHIK	1377.76
470–486	TDTLEDLFPTTKIPNPR	1958.02
641–654	AFREEAIKFS EEQR	1739.87
649–660	FSEEQRFNNFLK	1558.77

Peptides in bold were sequenced with MS/MS on the doubly charged ion. MetOx refers to peptides with oxidized methionines.

**Table 4:** MALDI-MS results of Ku70

**Ku70**		
Amino acid residues		[M+h]+
**101–115**	**NIYVLQELDN PGAKR**	**1729.92**
115–129	RILELDQFKGQQGQK	1787.06
116–129	ILELDQFKGQQGQK	1631.86
116–130	ILELDQFKGQQGQKR	1787.06
302–318	TFNTSTGGL LLPSDTKR	1807.96
326–339	QIILE KEETEELKR	1757.96
446–461	MPFTE KIMATPEQVG K	1838.90 (2 MetOx)
471–488	FTYRSDSFENPVLQQHFR	2271.09
**475–488**	**SDSFENPVLQQHFR**	**1703.81**

Peptides in bold were sequenced with MS/MS on the doubly charged ion. MetOx refers to peptides with oxidized methionines.
