# Supplementary figures and images for: Identification of poly(ADP-ribose)polymerase-1 and Ku70/Ku80 as transcriptional regulators of S100A9 gene expression
Source: BMC Mol Biol. 2006 Dec 22;7:48. doi: 10.1186/1471-2199-7-48 (PMC1766928; doi:10.1186/1471-2199-7-48)

## Slide 1
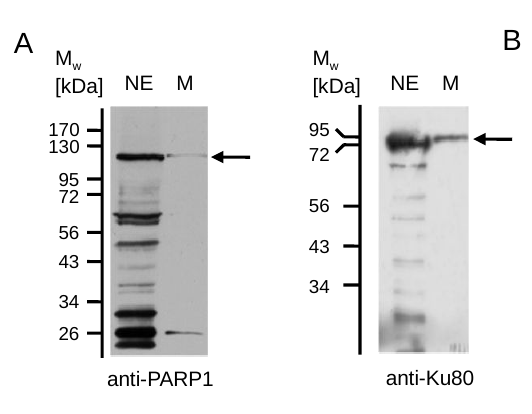

B
A
Mw
[kDa]
Mw
[kDa]
NE M
NE M
95
72
56
43
34
170
130
95
72
56
43
34
26
anti-Ku80
anti-PARP1

Supplement: Additional File 1 — Western Blot analysis. Aliquots of the Raji nuclear extract (NE) and the 1 M NaCl eluate of affinity chromatography (M) was subjected to SDS-PAGE. The Western-blot analysis was performed using PARP-1- and Ku80-specific antibodies. [file 1471-2199-7-48-S1.ppt]
